# Variations in obsessive compulsive disorder symptomatology across cultural dimensions

**DOI:** 10.3389/fpsyt.2024.1329748

**Published:** 2024-01-23

**Authors:** Wassim Hassan, Samer El Hayek, Renato de Filippis, Mario Eid, Sarah Hassan, Mohammadreza Shalbafan

**Affiliations:** ^1^ College of Medicine, University of Illinois, Chicago, IL, United States; ^2^ Medical Department, Erada Center for Treatment and Rehabilitation in Dubai, Dubai, United Arab Emirates; ^3^ Psychiatry Unit, Department of Health Sciences, University Magna Graecia of Catanzaro, Catanzaro, Italy; ^4^ Department of Psychiatry, Lebanese American University, Beirut, Lebanon; ^5^ The Chicago School of Professional Psychology, Chicago, IL, United States; ^6^ Mental Health Research Center, Psychosocial Health Research Institute, Department of Psychiatry, School of Medicine, Iran University of Medical Sciences, Tehran, Iran

**Keywords:** obsessive compulsive disorder, culture, psychiatry, mental health, social psychiatry

## Introduction

Obsessive-compulsive disorder (OCD) is a chronic mental health condition most commonly characterized by persistent, intrusive, unwanted, and distressing thoughts (obsessions) and repetitive behaviors or mental acts (compulsions) performed in an attempt to transiently alleviate preoccupation with these obsessions (1). Those suffering from OCD find it difficult to control these thoughts and behaviors, leading to significant distress and an impaired quality of life. The cycle of obsessions and compulsions can significantly impact various aspects of a person’s life, including work, school, relationships, and daily tasks. Although the standard public understanding of OCD is built around an obsession with cleanliness and order, other manifestations commonly include fear of physical harm, hoarding, repetition of words or phrases, and moral obsessions or rituals (commonly via the medium of religion). The exact cause of OCD is unknown, but it is believed to involve a combination of genetic, neurological, behavioral, cognitive, and environmental factors (2). OCD can often co-occur with other mental health disorders, such as anxiety disorders, depression, and eating disorders (3).

Common treatments that have shown promising efficacy in OCD include Exposure and Response Prevention (ERP), cognitive-behavioral therapy (CBT), and medications such as selective serotonin reuptake inhibitors (SSRIs) (4). The goals of therapy are to decrease the frequency and intensity of obsessions and compulsions, mitigate anxiety, and improve overall quality of life. OCD is a heterogeneous condition with a wide range of symptoms, and this heterogeneity often complicates a clinician’s ability to diagnose the disorder. However, the Yale-Brown Obsessive-Compulsive Scale (Y-BOCS) is a widely used tool that aids in the recognition and classification of different OCD subtypes (5). A meta-analysis of 21 studies utilizing the Y-BOCS checklist found that symptoms of OCD can be classified into four major categories: symmetry, forbidden thoughts, hoarding, and cleaning (6). Emerging evidence suggests that treatment response, heritability, disease course, and neuropsychological profile differ among these subtypes (7). While the core features of OCD remain consistent, cultural variations can influence the expression (**Table 1**), interpretation, and management of the disorder. It is widely accepted that culture has an impact on the clinical presentation of OCD. However, literature comparing OCD manifestations across cultures in a standardized approach is scarce (8). In addition, although OCD is a universal phenomenon, research into the disorder remains mostly limited to Europe and North America (9).

**Table 1:** Common cultural associations with specific types of OCD compulsions.

Culture	Common Compulsion Type
Middle Eastern	Religious and Ritual Compulsions
South American	Aggressive or Violent Compulsions
East Asian	Symmetry and Order Compulsions
Indian Subcontinent	Contamination and Cleaning Compulsions
African	Superstitious Compulsions
Western	Checking and Counting Compulsions

Culture can affect the content of obsessions as well as the expression of compulsions (10). For example, patients from different backgrounds may experience obsessions related to culturally specific taboos, fears, or religious themes. Compulsions can take on the form of religious rituals or superstitious behaviors, which may be challenging to identify as these behaviors are often encouraged within the cultural context (10). For this reason, more research on cross-cultural manifestations of OCD is needed, as current clinical tools may fail to identify the disorder in patients exhibiting acceptable behaviors within their specific cultural context. Mental health professionals should also recognize the significance of the connection between OCD and culture and take the sociocultural context into account during their clinical evaluation. In this opinion letter, we provide a condensed overview of the available literature concerning the intersection of OCD and culture and emphasize the need to customize interventions by factoring in this pertinent aspect.

## Current literature on OCD and cultural considerations

Research into the extent and the mechanisms through which culture affects the clinical presentation of OCD remains scarce, but the available literature on this topic provides valuable insights that can direct the efforts of future research. Vismara and colleagues present, in their recently published article, a relevant overview of the different sociodemographic and clinical features of patients with OCD and highlight the intricate role of the cultural context (11).

A study conducted by Fontenelle et al. in 2004 assessed the clinical presentation of 101 adult patients with OCD in Brazil and compared them with 15 clinical samples from North and Latin America, Europe, Africa, and Asia identified through a systematic review (12). Results showed that, universally, patients with OCD are predominantly females with an early age of onset. The authors reported a predominance of aggressive obsessions among Brazilian patients, compared to a predominance of religious obsessions among Middle Eastern patients (12). These results echo other studies that found that OCD symptomatology in South American countries often involves themes of aggression (13). They also affirm studies conducted in the Middle East which found a larger proportion of religious obsessions and compulsions compared to Western societies (14).

Another study conducted in 2009 by Yorulmaz and colleagues comparing OCD presentations between Turkish and Canadian samples found that the type of thought control varied among cultures (15). In particular, Turkish patients utilized thought suppression while Canadians more frequently used self-punishment (15). A separate study performed by the same authors compared Muslim and Christian individuals from Turkey and Canada, respectively. Results showed that Muslim participants scored higher on OCD symptoms and reported more concerns about their unwanted thoughts. The authors also found that individuals with higher religiosity, regardless of religion category, reported experiencing more obsessional thoughts and checking behaviors, supporting a longstanding hypothesis that a hyper-fixation on religion may be contributive to the pathology of OCD (16).

A recent study, the largest cross-cultural comparison to date in the field of OCD, compared 1,187 adults with OCD from Brazil (n = 951) and the United States (n = 236) (17). Both groups shared a higher female-to-male ratio as well as a similar age of onset, likely reflecting specific biological processes underlying the pathophysiology of OCD. The groups differed, however, in their comorbidities. Patients with OCD in the United States were significantly more likely to have comorbid alcohol and substance use disorders, while those in Brazil were more likely to have comorbid anxiety and posttraumatic stress disorder. The authors suggested that this difference is due to cultural or environmental factors (17).

Non-Western societies and groups have not received widespread attention in the context of OCD research. Wetterneck et al. found that only 24% of studies on OCD symptomatology emerging in the United States and Canada included Latino participants (18). Additionally, although African Americans suffer from OCD at a similar rate as the general population (19), they represent less than 2% of all participants in OCD studies (9). In a study analyzing 21 trials from 1995 to 2008, findings indicated that only 1.6% of study participants identified as Asian (9). In Iran, a recent systematic review identified religious misconceptions and family relationships as major influences on OCD symptomatology (20). Reddy et al. reported that by 2010, there had only been one epidemiological study focused on Indians with OCD (21). In brief, there is limited research regarding minority demographics across racial, ethnic, and religious categories when it comes to the understanding of the symptomatology and treatment of OCD.

One should keep in mind specific culture-bound presentations that fall under the category of Other Specified Obsessive-Compulsive and Related Disorders, as discussed in the Diagnostic and Statistical Manual of Mental Disorders (DSM-5-TR). These include *Jikoshu-kyofu* and *Shubo-kyofu* in Japan, olfactory reference disorder (related specifically to the *Jikoshu-kyofu* variant but seen in various cultures other than Japan), and *Koro* in Southeast Asia (22).

## Cultural considerations in treatment

Shifting gears, Abed and Pauw proposed an evolutionary psychology framework that the origins of OCD lie within a mental model of natural risk assessment in the human brain (23). In other words, an individual embedded in a particular cultural environment may perceive certain threats as more readily imminent than others, leading to the development of an obsessive attempt to avoid danger, ultimately resulting in OCD. This model may explain the prevalence of themes of aggression in South America, wherein many countries have experienced a dramatic rise in violence in recent years, as well as the themes of religious obsessions and compulsions in the Middle East and North Africa region, where the population generally exhibits more religiosity than other parts of the world (23).

In the context of religion, it is important to recognize the significance of early intervention in managing OCD. Early identification and treatment of OCD are strong predictors of treatment success. Indeed, numerous studies attested to more favorable outcomes in people who begin treatment in childhood as opposed to adulthood (24). From this perspective, understanding the variations of OCD symptomatology among religious groups can be crucial in promoting a knowledgeable population that is better equipped to identify and seek early treatment. For example, in some religions, the frequent use of sacred phrases and repeated performance of religious rituals are viewed as signs of devotion and piety. This may lead to a positive social response to an individual who might be displaying OCD symptoms, albeit in a manner considered socially acceptable and even encouraged. In this context, Religion-Adapted Cognitive Behavioral Therapy (R-CBT) has been widely recognized to be just as efficacious as secular CBT, with some studies even reporting findings that suggest it has greater success (25). In many populations, religious beliefs form the foundation of the paradigm through which individuals understand themselves, the world, the metaphysical, and even their illnesses. Paradoxically, an individual’s relationship with a particular religion may be the root from which their pathology has grown. Thus, integrating religious literacy into CBT, where relevant, may assist patients in finding a balance between the role of religion in their lives and the symptomatology of their OCD, ultimately improving their quality of life without necessitating the neglect of core tenets in their journey towards health.

The role of parenting is also often under-emphasized in not only the identification of OCD, but also the progression and subsequent treatment course (or lack thereof). More recent studies have found that cultural friction and assumptions from the surrounding community play a non-negligible factor from the perspective of parents with children suffering from OCD (26). Once treatment is sought, outcomes are generally favorable. However, there remains a massive hill to climb in order to reach the point in which treatment becomes a consideration in many cultures. A culturally literate provider will understand these insidious obstacles and is naturally more equipped to propose solutions in a manner palatable to the audience in question.

## Conclusion and future directions

Although the current literature on this topic is limited, it is evident that cultural norms, beliefs, and values can significantly influence the experience of OCD, as well as the interpretation of symptoms by affected individuals, their communities, and mental health professionals. The latter continues to misidentify OCD symptoms, highlighting the need for further education and training in the field (27). Clinicians should approach OCD, as well as mental health in general, by taking into consideration the sociocultural context of the patient. To do this effectively, mental health professionals should conduct culturally sensitive assessments that account for the unique cultural factors influencing the presentation of OCD. Culturally competent clinical practice is not only an ethical responsibility but also a fundamental requirement for providing effective and patient-centered care to individuals from diverse backgrounds.

It is also crucial to acknowledge the need for further research in this field. While strides have been made in recognizing the cultural dimensions of OCD, much work remains to be done. The body of knowledge related to the interplay between culture and OCD is continually expanding, and future research endeavors should aim to delve deeper into the specific cultural factors that influence the course of the disorder. Additionally, research should aim to develop culturally-adapted interventions and treatment strategies that respect cultural values and beliefs while offering evidence-based care.

## Author contributions

WH: Conceptualization, Data curation, Writing – original draft. SE: Conceptualization, Supervision, Writing – original draft. Rd: Conceptualization, Writing – review & editing. ME: Writing – review & editing. SH: Writing – review & editing. MS: Conceptualization, Supervision, Writing – review & editing.
